# The Phylacogen Treatment of Infections

**Published:** 1914-02

**Authors:** 


### The Phylacogen Treatment of Infections.

An interesting experience with phylacogens has been narrated by Dr. E. H. Troy, of Oklahoma. It appeared in a recent number of the *International Journal of Surgery.*

"I have treated twenty-four cases of rheumatism," writes Dr. Troy. "Their recoveries were as rapid as remarkable. One man of 32 had had rheumatism for three years; he was confined to bed for three months, and eight months elapsed before he was able to work. He was brought to the hospital on a bed and had to be lifted on a sheet. I gave him one done of phylacogen daily, and in six days he walked to the station, carrying his suit case. Another patient, a man 24 years old, had inflammatory rheumatism when 10 years of age. He was confined to bed for six months. He has suffered all his life, and had visited the various watering-places in America, receiving very little benefit. The last four years he had been almost incapacitated. I gave him ten doses of phylacogen, and his recovery was rapid."

Dr. Troy refers to a number of other cases of infection, including chronic otitis media, sycosis, acne, carbuncle and erysipelas, in the treatment of which he has been singularly successful, and adds:

"The administration of phylacogen is peculiarly adapted for the treatment of infectious diseases. * * * The only requirement is to make a diagnosis. If you are treating infectious diseases without making a diagnosis, however, do not be disappointed if you do not get results with the phylacogens."

